# Effect of physiotherapy on the promotion of bone mineralization in preterm infants: a randomized controlled trial

**DOI:** 10.1038/s41598-022-15810-6

**Published:** 2022-07-08

**Authors:** Galaad Torró-Ferrero, Francisco Javier Fernández-Rego, Juan José Agüera-Arenas, Antonia Gomez-Conesa

**Affiliations:** 1 International School of Doctorate of the University of Murcia (EIDUM), University of Murcia, Murcia, Spain; 2 Department of Physical Therapy, Faculty of Medicine, University of Murcia, 30100 Espinardo-Murcia, Murcia, Spain; 3 Neonatal Intensive Care Unit, Clinic Hospital University Virgen de La Arrixaca, Murcia, Spain; 4 Research Group Research Methods and Evaluation in Social Sciences, Mare Nostrum Campus of International Excellence, University of Murcia, Murcia, Spain

**Keywords:** Diseases, Metabolic disorders, Bone, Paediatric research, Disease prevention, Rehabilitation

## Abstract

Preterm infants have a low level of bone mineralization compared to those born at term. The purpose of the present study was to investigate the effect of reflex locomotion therapy (RLT) on bone mineralization and growth in preterm infants and compare its effect to other physiotherapy procedures. Forty-six preterm infants born at 29–34 weeks were randomized into three groups: one group received RLT (n = 17); the other group received passive movements with gentle joint compression (n = 14); and the control group received massages (n = 15). All the treatments were performed at the neonatal unit for one month. The main outcome measure was bone mineralization, which was measured using the tibial speed of sound (Tibial-SOS). All the groups were similar in terms of gestational age (31.8 ± 1.18), birth weight (1,583.41 ± 311.9), and Tibia-SOS (1,604.7 ± 27.9) at the beginning of the intervention. At the end of the study, significant differences were found among the groups in the Tibial-SOS [F(4,86) = 2.77, p = 0.049, η_p_^2^ = 0.114] in terms of the benefit to the RLT group. In conclusion, RLT has been effective at improving Tibial-SOS levels and has been more effective than other physical therapy modalities; therefore, it could be considered an effective physiotherapeutic modality for the prevention and treatment of osteopenia from prematurity.

## Introduction

Osteopenia of prematurity is characterized by a reduction in bone mineral content. It is multifactorial, progressive and has variable severity^1,2^. It appears in 30% of children under 1500 g and occurs in more than 50% of new-borns weighing less than 1000 g^3^.

Postnatal bone mineralization in preterm infants is significantly behind the expected intrauterine bone mineralization^4^; in addition, a poor mineralization rate is maintained in children and young adults who are born prematurely^5,6^, which leads to reductions in peak bone mass, weaker bones, shorter heights, and higher fracture rates compared to term infants^7^.

Mechanical stress is one of the most stimulating factors for bone formation and growth, and while physical activity increases bone mass in children, adolescents and adults^8–10^, inactivity leads to bone resorption and a decrease in bone mineral density^10,11^.

Physiotherapy modalities have shown effectiveness in treating osteopenia in preterm infants, obtaining favourable results when applying passive movements with gentle compression^12–16^.

Based on data from the adult population, active versus passive mobilizations are more effective for the prevention of osteopenia^17^; however, to develop active movement in preterm infants, methods that do not require their active participation are necessary. Therefore, reflex locomotion therapy (RLT)^18^ may be a suitable method of choice.

RLT consists of stimulation of the central nervous system (CNS) by proprioceptive stimulation, which activates innate locomotion patterns. This stimulation provokes a reaction that involves the whole body, producing synergistic muscle contractions, which leads to specific active and involuntary movement patterns^18–21^. Through RLT, CNS activation takes place from the spinal level to the subcortical and cortical areas^20,21^.

Taking all this into account, we set out to investigate the effect of active-resisted mobilizations that are triggered by proprioceptive stimulation from RLT on bone mineralization and growth in preterm infants and to compare their effect against another passive modality of physiotherapy that has previously been shown to be effective^12–16^.

## Results

**Figure 1:**
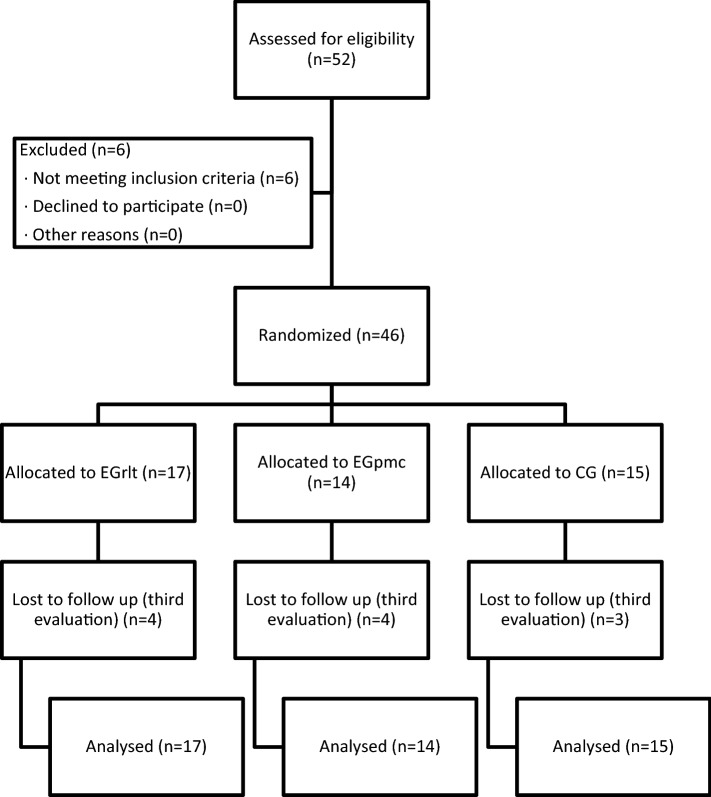
Participant flow diagram.

Fifty-two preterm infants admitted to Virgen de la Arrixaca Clinical University Hospital (HUCVA) were selected to be included in this study (Fig. 1) from February 2016 to July 2020. Of them, 6 were discarded because they met the exclusion criteria prior to randomization: one of them developed an intraventricular haemorrhage grade IV, two developed necrotizing enterocolitis that required surgical intervention, and the remaining were maintained on mechanical ventilation when receiving full enteral nutrition. The 46 infants were randomized into three groups (EGrlt: 17, Egpmc: 14, and CG: 15). Of the 46 preterm infants who started the study, 11 (23.91%) could not be subjected to the last measurement (4 from the Egrlt, 4 from the Egpmc and 3 from the CG), because they were discharged prior to the end of the 4 weeks of scheduled treatment (Fig. 1).

The three groups were similar in terms of GA (M = 31.8; SD = 1.18; p = 0.75), birth weight (M = 1,583.41; SD = 311.9; p = 0.247) and birth weight Z score (M = − 0.29; SD = 0.93; p = 0.084), as a group of very preterm infants with low birth weights. The groups were also similar in terms of age (M = 33.5; SD = 1.24; p = 0.689) and Tibial-SOS (M = 1604.7; SD = 27.9; p = 0.706) at the beginning of the intervention; however, significant differences were found regarding sex (p = 0.000; female in Egrlt = 58.82%; and Egpmc = 50%; CG = 40%) (Table 1).

**Table 1:** Baseline data.

	EGrlt (n = 17)	EGpmc (n = 14)	CG (n = 15)
Gestational age (weeks)	31.76 ± 1.41	31.99 ± 1.36	31.66 ± 0.65
Birth weight (g)	1,508.24 ± 321.5	1,695 ± 404.65	1,564.47 ± 151.06
Birth weight Z score	− 1.33 ± 0.23	− 0.49 ± 0.26	− 0.78 ± 0.25
Gender (female)*	10 (58%)	7 (50%)	6 (40%)
Gestational age at the beginning of the intervention (weeks)	33.45 ± 1.33	33.74 ± 1.47	33.34 ± 0.89
Weight at the beginning of the intervention (g)*	1,588.59 ± 247.23	1,965.14 ± 449.32*	1,653.6 ± 138.23
Height at the beginning of the intervention (cm)	41.37 ± 1.99	43.96 ± 2.99	42.9 ± 1.20
Head circumference at the beginning of the intervention (cm)	29.06 ± 1.22	30.71 ± 1.73	29.6 ± 1.14
Tibial speed of sound at the beginning of the intervention (m/s)	1,608.06 ± 29.73	1,599.64 ± 29.3	1,605.6 ± 25.62

*p < 0.05 for differences among groups. CG = Control Group. EGpmc = Experimental group treated with passive movements with gentle compression. EGrlt = Experimental group treated with reflex locomotion therapy. Data are presented as means ± SD.

No adverse effects were detected in the participants as a consequence of the different types of treatments. Moreover, none of the infants in the present study were diagnosed with osteopenia at baseline or during the intervention period or presented any fractures.

### Tibial speed of sound

Regarding the Tibial-SOS measurements, significant differences were observed among the different types of interventions regarding the different moments of measurement F (2,86) = 40.42, p < 0.001, η^2^ = 0.223], the values obtained for the different groups [F (2,43) = 3.64, p = 0.034, η^2^ = 0.105] and the interaction of the variable in the different groups [F (4,86) = 2.77, p = 0.049, η^2^ = 0.038].

**Figure 2:**
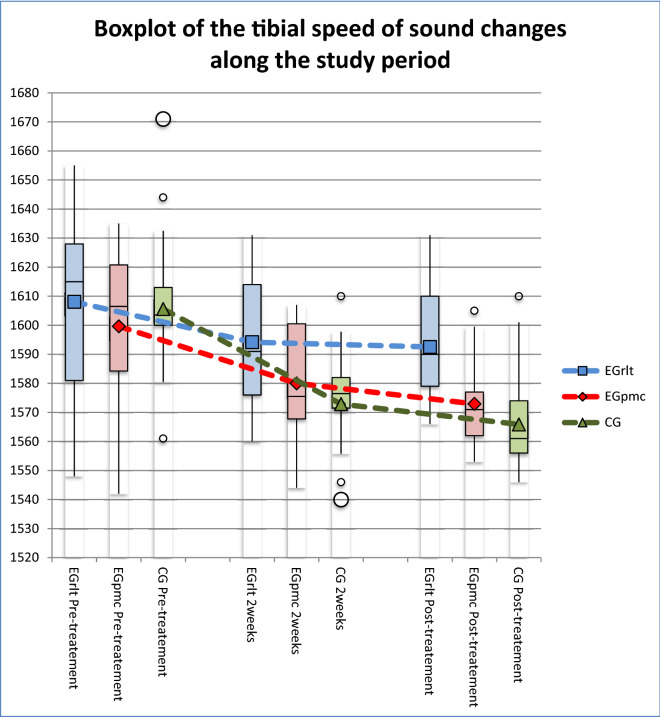
Box plot of the tibial speed of sound changes during the study period.

The results show that there are differences among the groups regarding how they evolve at the different moments of measurement of the Tibial-SOS, and if we look at Fig. 2, we can see that the group with the best evolution is EGrlt, and the group with the worst evolution is CG.

The results among the groups and the different moments of measurement showed significant differences in the Tibial-SOS between the infants who received RLT compared to those who received massage at two weeks after starting treatment, with better EGrlt results (p = 0.016; 95% CI 6.607–36.01). At the end of the treatment, the EGrlt group showed significantly better Tibial-SOS values than the EGpmc (p = 0.015; 95% CI 5.399–33.8) and CG groups (p = 0.001; 95% CI 12.859–40.47). There were no meaningful differences in the Tibial-SOS values between the group treated with PMC and the group treated with massage both in the second evaluation (p = 0.729; 95% CI − 6.891 to 21.15) and in the third (P = 0.666; CI95% = − 5.452 to 19.58) (Table 2, Fig. 2).

**Table 2:** Effect of the different interventions on the tibial speed of sound (m/s) and anthropometric measures during the study period.

	EGrlt (Mean ± SD)	EGpmc (Mean ± SD)	CG (Mean ± SD)	Pairwise comparison	Mean difference	95% CI	
						Lower bound	Upper bound
**Tibial-SOS (m/s)**							
Pre-treatment	1,608.06 ± 29.73	1,599.64 ± 29.3	1,605.6 ± 25.62	EGrlt = EGpmc	8.42	− 13.39	30.22
				EGrlt = CG	2.46	− 17.72	22.63
				EGpmc = CG	− 5.96	− 26.89	14.97
2 weeks	1,594.18 ± 23.47	1,580 ± 20.67	1,572.87 ± 15.99	EGrlt = Egpmc	14.18	− 22.51	30.60
				EGrlt > CG*	21.31	6.61	36.01
				EGpmc = CG	7.13	− 6.89	21.16
Post-treatment	1,592.53 ± 21.2	1,572.93 ± 16.51	1,565.87 ± 16.32	EGrlt > EGpmc*	19.60	5.40	33.80
				EGrlt > CG*	26.66	12.86	40.47
				EGpmc = CG	7.06	− 5.45	19.58
**Weight (g)**							
Pre-treatment	1,588.59 ± 247.23	1,965.14 ± 449.32	1,653.6 ± 138.23	EGrlt < EGpmc*	− 348.32	− 752.00	− 80.00
				EGrlt = CG	− 150.00	− 260.00	− 20.00
				EGpmc > CG*	230.94	− 75.00	570.00
2 weeks	2,007.82 ± 286.97	2,431.79 ± 501.62	2,256.73 ± 229.87	EGrlt < EGpmc*	− 485.34	− 1030.00	− 50.00
				EGrlt = CG	− 296.45	− 490.00	− 130.00
				EGpmc = CG	201.53	− 270.00	710.00
Post-treatment	2,302.94 ± 331.11	2,726.07 ± 510.05	2,598.07 ± 396.25	EGrlt < EGpmc*	− 635.11	− 1029.40	− 240.80
				EGrlt = CG	− 552.75	− 851.50	− 254.00
				EGpmc = CG	82.36	− 355.10	519.80
**Height (cm)**							
Pre-treatment	41.37 ± 1.99	43.96 ± 2.99	42.9 ± 1.2	EGrlt < EGpmc*	− 5.00	− 7.00	− 0.50
				EGrlt = CG	− 2.00	− 3.50	0.00
				EGpmc = CG	2.00	− 1.00	5.00
2 weeks	44.59 ± 3.29	46.04 ± 2.48	45.73 ± 1.96	EGrlt = Egpmc	− 1.63	− 4.72	1.47
				EGrlt = CG	− 0.88	− 3.97	2.22
				EGpmc = CG	0.75	− 1.64	3.14
Post-treatment	45.85 ± 2.69	47.18 ± 2.84	46.53 ± 2.05	EGrlt = Egpmc	− 2.08	− 4.31	0.16
				EGrlt = CG	− 0.45	− 2.56	1.66
				EGpmc = CG	1.63	− 0.90	4.14
**Head circumference (cm)**							
Pre-treatment	29.06 ± 1.22	30.71 ± 1.73	29.6 ± 1.14	EGrlt < EGpmc*	− 1.76	− 3.90	0.40
				EGrlt = CG	− 0.53	− 1.94	0.89
				EGpmc = CG	1.23	− 0.51	2.98
2 weeks	30.88 ± 1.36	32.36 ± 1.97	30.93 ± 2.43	EGrlt = Egpmc	− 1.11	− 3.78	1.55
				EGrlt = CG	− 0.34	− 2.19	1.51
				EGpmc = CG	0.78	− 1.30	2.86
Post-treatment	32.06 ± 1.34	33.43 ± 1.79	32.1 ± 2.87	EGrlt = Egpmc	− 1.67	− 3.58	0.24
				EGrlt = CG	− 1.10	− 2.96	0.76
				EGpmc = CG	0.57	− 1.02	2.16

*p < 0.05 for differences among groups. CG = Control Group. EGpmc = Experimental group treated with passive movements with gentle compression. EGrlt = Experimental group treated with reflex locomotion therapy. 95% CI = 95% Confidence Interval. Data are presented as means ± SD.

Lastly, we can observe that all the groups significantly lost bone mineralization between the first and third measurements (EGrlt: p = 0.043, 95% CI 0.355–30.704; EGpmc: p = 0.001, 95% CI 9.993–43.436; CG: p < 0.001, 95% CI 23.579–55.888), but this loss was less pronounced in the EGrlt, since in this group, there were no significant differences between the first and second measurements (p = 0.069; 95% CI − 0.815 to 28.581) or between the second and third measurements (p = 0.950; 95% CI − 6.816 to 10.11). In contrast, in the other two groups, we did observe significant losses between the first and second measurements (EGpmc: p = 0.013, 95% CI 3.446–35.84; CG: p < 0.001, 95% CI 17.085–48.381).

### Anthropometric measurements

**Figure 3:**
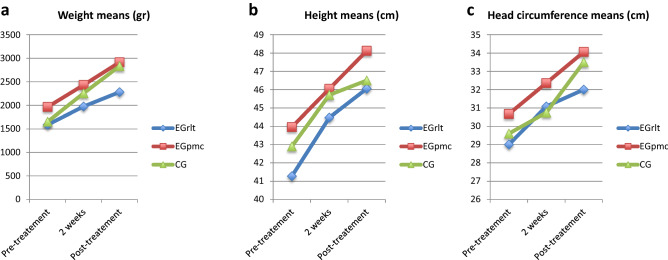
Effect of the intervention on the anthropometric measures of weight (**a**), height (**b**) and head circumference (**c**).

Regarding weight, significant differences were observed among the different moments of measurement [F (2,10.535) = 197.30, p < 0.001, η^2^ = 0.628] and regarding the groups [F (2,9.057) = 8.617, p = 0.008, η^2^ = 0.346] but not for the variable interaction [F (4,8.81) = 1.601, p = 1.6, η^2^ = 0.046]. With regard to height, differences were found among the measurement moments [F (2,11.394) = 82.073, p < 0.001, η2 = 0.402] but not among the groups [F (2,8.556) = 1.985, p = 0.196, η^2^ = 0.173] or regarding the interaction of the variable [F (4,9.359) = 1.753, p = 0.22, η^2^ = 0.035]. Lastly, regarding head circumference, differences were found for the moments of measurement [F (2,34) = 148.027, p < 0.001, η2 = 0.543] but not regarding the group [F (2,17) = 1.605, p = 0.23, η^2^ = 0.14] or the interaction [F (4,34) = 0.827, p = 0.517, η^2^ = 0.013] (Table 2, Fig. 3).

Regarding the Z scores, no differences were observed in terms of the interaction of these variables on the group with respect to weight [F (4.86) = 1.541, p = 0.215, η^2^ = 0.067], height [F (4, 86) = 1.068, p = 0.354, η^2^ = 0.047] and head circumference [F (4.86) = 0.482, p = 0.697, η^2^ = 0.022].

When analysing the results among the different groups and the different moments of measurement in relation to weight, height and head circumference, we found that all the groups showed statistically significant improvements in weight, height and head circumference after each measurement compared to the previous measurement. If we observe the graphs (Fig. 3), the group that evolves the best in weight gain is the CG, for height it is the EGrlt and regarding head circumference, all groups evolved in a similar way.

At the beginning of the study, the weight in the EGpmc group was significantly higher than the weight observed in the EGrlt group (p = 0.034; 95% CI − 752 to − 80). Regarding the second measurement, which was performed two weeks after treatment was started, there were no longer differences in weight between the groups treated with PMC and RLT (p = 0.091; 95% CI − 1030 to − 50), but there were differences between the CG and EGrlt groups (p = 0.02, 95% CI − 490 to − 130). At the end of the treatment, we found differences in weight in favour of EGpmc (p = 0.0025; 95% CI − 1029.4 to − 240.8) and CG (p = 0.011; 95% CI − 355.1 to 519.8) with respect to the EGrlt.

Despite these differences, because no differences were found for the interaction of the variables, we can conclude that all the groups evolved equally in terms of weight gain, height and head circumferences.

### Harms

There were no adverse events reported due to intervention in any of the three groups.

## Discussion

The expected sample size was 66 preterm infants, with 22 for each group; however, due to sepsis in the hospital's NICU, the unit was forced to close. No more patients were admitted, and ongoing studies had to be postponed. This situation led us to reach the scheduled closing date, so the study had to end. Therefore, the total sample size was 69.69% of the total sample size (46 new-borns distributed over three study groups), showing a significant difference in relation to the intervention studied.

The main objective of this research is to verify the effect of RLT, which is understood as non-voluntary active-resisted mobilizations, on bone mineralization and growth in preterm infants and to compare its effect with those of other passive physiotherapy modalities.

In this regard, the outcome of this study indicates that RLT has a positive effect on bone mineralization when measured with quantitative ultrasound (QUS), consisting of a reduction in the drop in bone mineralization characteristic of preterm infants that can lead to osteopenia.

In vitro studies have shown that forearm quantitative US variables correlate significantly with bone strength, which corresponds to bone mineral assessment by DXA in children^22^.

A high correlation between dual energy X-ray absorptiometry (DXA) and QUS has also been observed in adults to assess bone quality^23^ and osteoporosis^24^. In preterm infants and new-borns, QUS also provides information on bone mineralization and density, elasticity, cortical thickness, and bone microarchitecture^25^. Therefore, QUS can be considered an appropriate tool for assessing osteopenia in preterm infants^26,27^.

When comparing the three treatments performed here, the modality with RLT showed better results for the Tibial-SOS values with respect to the group treated with PMC and the massage group (control), showing a small effect size; however, no significant differences were found between the group treated with PMC and the control group. This result contrasts with the results obtained by other authors, which highlighted the positive effect of PMC compared to control groups to which a placebo was not applied^28–30^, although in one of them, the times and frequency of PMC treatment were greater^29^. In our study, consistent with other authors^31–34^, the results show that massage has no impact on bone mineralization.

Despite the energy expenditure that RLT treatment could entail for the baby, and considering that the baby performs active-resisted work, there has been no negative effect in relation to measures of weight, height and head circumference, since, as the data showed, all the groups evolved equally in terms of anthropometric variables. Anthropometric measurements were also not diminished in children in the EGpmc and CG groups, similar to other studies performed on low birth weight babies treated with PMC compared with controls without treatment or a placebo with massage^28,30,32,34^, in which, as in our study, no significant differences were found among the study groups in relation to weight, height, and head circumference. These results are supported by the Z scores, which show no differences among the groups.

In the present study, the EGrlt group started from a point of significant disadvantage regarding weight, height and head circumference compared to the EGpmc (Table 2), which is related to worse results for QUS values in preterm infants^26^. Therefore, it would be expected that this group’s evolution in terms of Tibial-SOS values would also be lower, and yet this was not the case. Although infants treated with RLT showed a lower weight than the group treated with PMC, the EGrlt significantly increased their weight in each measure, showing a gain similar to those of the remaining groups, since no differences were observed in terms of interaction (Fig. 3) and they ended showing better improvements in QUS. An explanatory hypothesis of this result may be due to the consideration of RLT as a non-voluntary active exercise, taking into account the results of different meta-analyses that relate active physical exercise and weight control, which, although performed in the paediatric population, could be extrapolated to the neonatal population^35,36^.

The weight gain in the CG of our study is consistent with the findings of other authors, who showed weight gain in preterm infants who received massage^37–40^, and in contrast to Eliakim et al. 2002^31^, who found weight improvements in favour of PMC compared to caresses and tactile stimulation.

Some limitations of our trial should be noted. The heterogeneity of the preterm infant risk factors could influence the results obtained here. Another limitation is the lack of long-term follow-up to determine the effect of the interventions on the Tibial-SOS.

It would be advisable to perform multicentre clinical trials with a large sample of preterm infants that guarantee the observation of the intervention effect at different gestational ages. It would also be convenient to perform studies with long-term follow-ups, in which the evolution of these children in early childhood and adolescence could be observed.

We can conclude that RLT has been effective in improving Tibial-SOS values, which may have a positive effect on the prevention of osteopenia in preterm infants. Furthermore, RLT has been shown to be more effective than other physical therapy modalities, such as PMC and massage, in improving the Tibial-SOS.

Due to the characteristics of the sample on which the intervention was performed, the results indicate that treatment with RLT is effective at improving bone mineralization in healthy preterm infants, and it could be considered an effective treatment to prevent osteopenia in this population.

## Methods

### Trial design

This prospective randomized clinical trial was performed in the neonatal service department of the HUCVA; from February 2016 to July 2020, 46 preterm infants participated in it and were randomized into three intervention groups, two treatment groups and a control group. This study was approved by the HUCVA ethical committee for clinical research, using all the procedures stipulated in the Declaration of Helsinki^41^. This study has been registered at ClinicalTrials.gov under identification number NCT04356807.

### Participants

The participants in this study were preterm infants born between the 29th and the 34th + 2 weeks of gestational age (GA) who were admitted to the neonatal unit, with haemodynamic stability and full enteral nutrition, whose parents or guardians signed the consent form authorizing the participation of their infant in this study. New-borns with neurological disorders, a need for mechanical ventilation, bronchopulmonary dysplasia, congenital malformations, metabolic diseases, genetic diseases, intraventricular haemorrhage III-IV, those taking diuretic or corticosteroid medication, and those with bone fractures at the time of randomization were excluded.

### Interventions

The participants in this study were divided into three groups that, along with standard nursing care, received different physiotherapy treatments.

The control group (CG) was given limb and core massage, with gentle deep pressures and caresses lasting 15 min a day in a single physiotherapy session, 5 days per week, for 4 weeks, which was considered a placebo since this intervention has no influence on bone mineralization^30–34,42^.

The experimental group (EGpmc) received passive movements with gentle compression (PMC) as described by Moyer-Mileur et al.^14^ and with the adaptations of Vignochi et al.^12^ in a 15-min physiotherapy session, 5 days per week for 4 weeks. These mobilizations consist of flexion and extension movements in all the joints of both the upper and lower extremities and ending with chest movements following the baby's respiratory pace.

The experimental group (EGrlt) received RLT according to the procedures used by other authors^18–21^ for 16 min, as divided into two physiotherapy sessions of 8 min each, 5 days per week for 4 weeks. The exercises corresponding to the motor complexes of the 1st phase of reflex rolling and reflex creeping was performed, by dedicating one minute to each side and performing two repetitions in each session.

For the 1st phase of reflex rolling, the child is placed in dorsal decubitus, with the head turned to one side at an angle of 30°, the spine as aligned as possible, and the limbs relaxed. The physiotherapist applies gentle pressure with his thumb at the point of intersection of the mammillary line with the diaphragm, between the 6th–7th intercostal space, in the hemithorax and on the side towards which the head rotates, with a dorsal-medial-cranial direction, while resisting the turning of the head towards the other side with the other hand ^18,19,21^.

For reflex creeping, the child is placed proned, then passively bringing the head to axial neck extension and 30 degrees of rotation. The upper limb, on the side on which the head is turned, is placed in a position of shoulder flexion between 120 and 135 degrees, with 30 degrees of abduction, leaving the epitrochlea supported; the wrist is aligned with the shoulder, the forearm rests on the palmar face, and the longitudinal axis of the humerus points towards the vertex of the lumbosacral hinge. The opposite arm is placed relaxed and parallel to the longitudinal axis of the body.

The leg on the side toward which the child's head is turned is supported, extended and relaxed. The other leg is placed with the hip in external rotation and abduction, leaving the support on the internal condyle of the femur, the knee slightly flexed, and the heel aligned with the ischium. The stimulation is performed, with the index finger of one hand, on the lateral tuberosity of the calcaneus, in the ventral-cranial-medial direction of the leg opposite to the turn of the head, and with the index finger of the other hand, on the epitrochlea of the arm towards which the head is turned, in a dorsal-medial-cranial direction^20,21^.

All the treatments were applied by the same physiotherapist who had more than 5 years of experience, and the infants in the 3 groups were evaluated under the same conditions.

### Outcomes

Bone mineralization data and anthropometric measurements of weight, height, and head circumference were collected.

To measure bone mineralization, the tibial speed of sound (Tibial-SOS) was recorded using a quantitative ultrasound device (QUS) (*Sunlight Omnisense 7000*)^25,26,43^. It was measured on the third lower part of the left tibia, by keeping the knee bent at a 90-degree angle. The measurement point was made perpendicular to the direction of the bone. Three to five consecutive measurements were made, and the average was calculated to determine the Tibial-SOS (m/s).

Although DXA is the gold standard for determining bone density in older children and adults, some challenges arise when using this method in preterm infants, including movement artefacts, difficulty scanning small and sick infants^44^, high cost^22^ and the cumulative radiation dose and the stress of transport to and restraint in the unit^26^. QUS techniques, otherwise applied to peripheral sites, are safe, radiation-free, easy to use, portable and cost effective. These characteristics make them favourable for use in assessing bone status in preterm infants^25,26^.

Moreover, the Tibial-SOS provides information on bone mineralization and density, elasticity, cortical thickness, and bone microarchitecture^25^. In addition, its efficacy in evaluating the state of bones in preterm infants has been demonstrated^26^, and this, in addition to its high reproducibility and its non-invasive properties^43^, makes it an ideal instrument for our purposes.

Measurements of weight, height and head circumference were also collected. Body weight was measured by placing the baby naked on a digital scale (SECA), the height was measured as the distance from head to heel with a non-elastic tape, and head circumference was measured at its widest part between the eyebrows and the occiput, also using a non-elastic tape.

Tibial SOS measurements were performed three times by a neonatologist: one day before starting treatment sessions, after two weeks of treatment and at the end of treatment. Inter-rater reliability and intra-rater reliability were high, with intraclass correlation coefficients (ICCs) of 0.852 and 0.861, respectively.

Anthropometric measures were taken from one day before starting the treatment to one day after finishing it, and collecting them on alternating days, according to the nursing protocol, and they were performed by staff. For our analysis, we used those that coincided with the day the Tibial-SOS was measured, or, failing that, the last measurement made before that day.

The Z score was calculated for birth weight and for weight, height and head circumference at the different measurement moments, following the 2013 Fenton growth charts for this purpose^45^.

### Sample size calculation

The sample size was determined by considering a 5% significance level, 80% statistical power and a high magnitude effect size according to Cohen’s criteria^46^. The software program nQuery Advisor version 7.0^46,47^ gave us a previous sample size of 66 preterm infants (22 for each study group). However, due to sepsis that affected the hospital's neonatal intensive care unit (NICU), this unit was forced to close. No more patients were being admitted, and the studies that were in progress had to be postponed. This situation caused us to reach the scheduled end date, so the study had to be finished. Therefore, the total sample size was 46 new-borns distributed over the three study groups. The statistical analysis was performed with 70% of the total sample size, and a significant difference was observed in relation to the studied intervention.

### Randomization

The groups were formed by simple randomization. The randomization procedure consisted of sealed envelope labels containing a number for each group. A non-researcher drew a random number from the envelope each time a new patient was proposed for treatment and made the assignment. For ethical reasons, twins and triplets were assigned to the same group with the same number.

### Blinding

All the personnel who performed the measurement tests were external to the study and were blinded to which intervention group the patients belonged. Likewise, participants, family, and data analysts were also blinded. The physiotherapist who performed the treatments was blinded to the objectives of the study.

### Statistical methods

The qualitative baseline sex characteristics of the infants were compared using a crosstab, and a Chi-square test was performed for its analysis. The quantitative variables (gestational age, birth weight, birth weight Z score, gestational age at baseline, weight, height, head circumference, anthropometric Z scores, and Tibial-SOS at baseline) were analysed by one-way analysis of variance. A mixed repeated measures analysis of variance was performed to compare the effect of the intervention on anthropometric and Tibial-SOS measures and on anthropometric Z scores, using the time of measurement as the intra-subject factor and the treatment group as the inter-subject factor. For cases in which the homoscedasticity assumption was not met, a robust mixed repeated measures analysis of variance was performed. The 95% confidence intervals (95% CI) were adjusted by Bonferroni. A statistical analysis with intention to treat was performed for all variables using SPSS (Statistical Package for the Social Sciences) for Windows (v.22.0)^48^. The statistical significance was stipulated with p < 0.05. For the effect size, the eta square (η^2^) was calculated, considering a value of > 0.14 as high; moderate with values between 0.14 and 0.06; and small values between 0.06 and 0.01^46,49,50^. The data are presented as the means ± standard deviation.

### Ethics approval and consent to participate

The study was approved by the HUCVA ethical committee for clinical research. Reference number: 07/15.

### Informed consent

Informed consent was obtained from all subjects and/or their legal guardian(s).

### Consent for publication

Not applicable.

### Registration

Trial registered at ClinicalTrials.gov. First posted date 22/04/2020. Registration number: NCT04356807. URL: https://clinicaltrials.gov/ct2/show/NCT04356807?cond=Physical+Therapy+to+Prevent+Osteopenia+in+Preterm+Infants&draw=2&rank=1.

## Data Availability

The full trial protocol can be found at ClinicalTrials.gov. The datasets used and/or analysed during the current study are available from the corresponding author on reasonable request. The pre-print version of this article is presented at https://www.researchsquare.com/article/rs-1037228/v1. This article is not published nor is it under publication elsewhere.
